# Pitch perfect: healthcare leadership lessons from Lionel Messi and the World Cup

**DOI:** 10.1017/ash.2026.10901

**Published:** 2026-10-07

**Authors:** Gonzalo Bearman

**Affiliations:** Infectious Diseases, Virginia Commonwealth University Health System, Richmond, USA

“Success is no accident. It is hard work, perseverance, learning, studying, sacrifice, and most of all, love of what you are doing or learning to do.”**—Pelé**

## Kickoff

In the wake of the 2026 World Cup, following weeks of anticipation, excitement, and {Citation}visit life and professional lessons through the lens of soccer, known affectionately as *O Jogo Bonito*-*The Beautiful Game* ^1^? In my case, as an Argentine-American, I grew up playing competitive soccer including 4 years at the NCAA Division I level and experienced many highs and lows along the way. I continue to play for a local club andenjoy the sport as a spectator. As both a soccer player and fan, I frequently observe parallels in soccer that mirror much of my experience as an infectious diseases (IDs) specialist and academic division chief. Although I previously explored soccer and leadership in medicine elsewhere, the themes are ongoing.^2^ Here I further explore lessons from soccer as a *school of life* and reflect on what sport has taught me about leadership, growth, collaboration, success, and remaining resilient in the face of ongoing challenges.

## What is leadership? From pitch side to the bedside

Much is published on leadership styles and strategies. In a soccer conference in Buenos Aires, Argentina, famed Spanish soccer manager Josep “Pep” Guardiola defined leadership simply as “a leader is defined as someone who makes others better.”^3^ In medicine, a clinical leader is someone who creates a better environment for colleagues, trainees, and staff. Improvement and growth can be measured in a variety of ways such as clinical services, academic, and educational accomplishments, promotions, and individual and team accolades. Perhaps more importantly, successful leadership and the pursuit of making others better is more accurately reflected in the ability to recruit, engage, and retain healthcare team members for the long term. Even if only 30% of team results are a function of level of engagement, 50%–70% of that engagement is driven by the management style of the leader, underscoring the importance of leadership for group and individual goals.^3^ In brief, leadership matters.

## The power of passion—necessary but not sufficient

High achieving soccer players must have selected qualities to succeed. Natural talent is a start, with appropriate strength, speed, agility, dexterity, and fitness. There must be a willingness to practice countless hours and to face the physical and psychological demands of practice and match play. But an important driver of success in sport and life is passion. Passion makes the hardships and sacrifices, or training, nutrition, psychological preparation, and intense match play tolerable, even enjoyable. Passion also fuels grit and longevity in careers. Lionel Messi and Cristiano Ronaldo are known for their lifelong passion for the sport, both reportedly finding immense joy in training, maintaining fitness, competition, and continually aiming for excellence and flow, a mental state in which one feels fully focused, energized, and performing at their best.^4^

Passion in medicine is evident in trainees and physicians who approach the practice of medicine as more than a job, always striving for excellence. Passion drives excellence in medicine and excellence is defined not only by medical knowledge, but by an ability to navigate the patient care system with skill and ongoing academic curiosity.^5^ Like match fitness, consult fitness in medicine, as explored elsewhere, is deliberate and intentional, and serves to maximize workflow, impact, and engagement.^5^ Passion in leadership is necessary to define individual and team purpose, provide clarity of goals, and inspire others to act.^6^ However, passion is not enough; leaders need compassion with an understanding of individual team member needs, values, and goals.^7^ As a division chair, I focus on understanding the unique qualities of each of my team members, to better understand them first as individuals, then as professionals, to better engage them in their professional journey.

## Lionel Messi’s success: a reminder of the importance of both talent and deliberate coaching

The 2026 World Cup was Lionel Messi’s 20^th^ year at the highest level of international soccer and a potent reminder of his evolution from youthful phenom to a top athlete and leader. Talent is defined as a natural skill or ability to be good at something, especially without being taught.^8^ Talent is necessary but not sufficient for success. Lionel Messi left his home of Rosario, Argentina at the age of 13 for La Masia, the Football Club Barcelona youth soccer academy. As the teenage Messi matured into a professional soccer player, with a debut in the Barcelona professional team at age 17, the coaching staff deliberately created a nourishing and mentoring atmosphere of training, nutrition, competition, and rest to maximize his development and minimize injury, fatigue, and burnout.^9^ Under this mentorship, the talented Messi further developed the key physical, mental, and leadership qualities required to excel as an elite athlete and team captain.^9^

Many young physicians complete their training with an abundance of talent, energy, and drive, willing to dive into patient care, administrative tasks, scholarly work, education, and other services to their home institutions. Excessive commitments will hinder professional growth and drive burnout, especially low value, uncompensated work.^10^ The role of a team leader is to coach and mentor young faculty so that work commitments are maximally focused, deliberate, and aligned with both individual and team goals.^10^ The work environment should also strive for reasonable work standards across all commitments, with explicit team coverage across colleagues to minimize fatigue and stress.^5^ The aim is for talent to thrive, if my team is not thriving, I am delinquent in my job.

## The great ones walk instead of run: why Lionel Messi walks more than runs, for longevity, rest

Part of the genius of Lionel Messi is that he grossly walks more than he runs on the pitch. This is not laziness, but rather a deliberate way of conserving energy, allowing him to read and interpret the game around him and to turn his thought into action at the most opportune moments.^11^ In fact, Lionel Messi’s mid match “zoning out” is a performance boosting brain hack, a neuroscience backed strategy for greater creativity and performance under times of stress.^12^ In conjunction with his disciplined workout regimen of varying intensities, sleep and recovery schedule, and consistently planned days off, Messi has effectively competed at the highest levels of professional soccer for over 20 years.

In the book *Rest: Why You Get More Done When You Work Less*, author Soojung-Kim Pang explores the concept of “deliberate rest”—an active, practiced skill that recharges cognitive energy, supercharges creativity, and prevents burnout.^13^ Most high-quality work is deliberate and focused and does not constitute most of the workday. The Four pillars of disciplined and deliberate productive rest are (1) microbreaks such as brief walks, naps or stopping mid task for enhanced creativity, (2) rigorous routines where more demanding tasks are tackled earlier, protected from digital distractions, (3) Deep play or active hobbies that are rewarding and stress free, and (4) scheduled sabbaticals or vacation days away from the office.^13^

For longevity in medicine, short of system level changes that improve work hours and minimize low value tasks, similar principles of deliberate rest and restorative actions apply. Unfortunately, while most physicians contractually are allowed 3–5 weeks of paid time off, Studies suggest that ∼60% take 15 or fewer vacation days annually, and over 70% still do patient care or electronic health record (EHR) work while on break.^14^ Drivers of this include heavy workload, heavy EMR demands and staffing, cross-coverage issues, and, likely, a sense of martyrdom. Further, many medical professionals overschedule their clinical and non-clinical work, resulting in escalating delays and frustrations during the workday.

Although the solutions for the above are beyond the scope of this manuscript, an essential first step is an awareness of excessive scheduling and cognitive load, without sufficient rest periods, either targeted during the workday, or as a schedule vacations. Potential strategies include minimizing administrative or other obligations on clinical days and prioritizing urgent tasks versus important tasks that can be later scheduled. Clinical leaders should explicitly engage teams on rest, recovery, and scheduling vacation periods with EMR cross-coverage by colleagues. More than ever, I strive to model the desired rest and recovery behaviors to represent the change I seek in colleagues.

## Halftime chat: the locker room and the conference room: more alike than you may think

According to World Cup champion Jorge Valdano, the power of the locker room is the power of the team.^3^ The success of the team manifests as a collective output, one that exceeds the individual talents of the team members. Success begins in the locker room, where shared discussions lead to a greater understanding of both team goals and individual demands. In this environment, group dynamics are the driving force, where the manager must perpetually foster team unity and ensure that all are proud of the collective goals, while understanding that the team consists of distinct individuals, with differing personalities and sensibilities. Team culture starts in the locker room.

I see the conference room in medicine as a similar touch point for team unity. It is in the conference room where I most regularly engage the team, where ideas and challenges are discussed and debated. The team dynamic, especially in the conference room, is critical. This is where Individuals must be heard and respected, where collective action is taken, and where leadership communicates important decisions. The *ethos* of the team is explicitly defined in the conference room and then applied to all matters or professional life.^10^

## No money, no mission: advocating for resources is frustrating, be prepared as nothing is guaranteed

In 2021, owing to economic pressures, Football Club Barcelona was forced to make difficult financial decisions. After 20 years at the club with unprecedented success, Lionel Messi’s contract was not renewed, and he was sold to French club Paris Saint Germain. This decision was not mutual, as the famed soccer player engaged in prolonged negotiations with club management with the intent to stay at Barcelona, even offering to accept a significant 50% reduction in salary.^9^ The negotiations between Messi and the club’s president and management were progressing but failed in the 23^rd^ hour owing to financial caps and structural obstacles posed by the Spanish League.^15^ Even for the iconic Lionel Messi, his unexpected, last-minute departure from Barcelona reflects the non-guarantee of contract negotiations in shifting financial environments.

Medicine, like professional soccer, is a business. Financial pressures may force a health system to make difficult decisions regarding salaries, resource management, and program expansion or contracture. Frontline provider and clinical leaders must be aware of these financial pressures and must be able to articulate their core strategic value to both patient care and health systems. Additionally, clinical leaders in medicine must be prepared for protracted, often frustrating negotiations with health system leaders and compensation plan managers. This requires an awareness of gaslighting by management and a honing of negotiation and persuasion skills to greenlight key initiatives.^16^ Even with preparation and persistence, as with sport, I have learned that the outcome is never guaranteed.

## Diversity strengthens teams and improves outcomes

World soccer is now more diverse than ever. The most competitive leagues in the world such as the English Premier League, Spain’s La Liga, the German Bundesliga and the Italian Serie A are replete with players of multiple nationalities and ethnicities. For example, the English Premier League has 60% + foreign (non-English) players.^17^ Even national teams are now less homogenous, with 50%–80% of storied teams such as France, Germany, and Spain comprised of players from different ethnicities, migrant or territorial backgrounds.^18–20^ Women’s soccer is the fastest growing team sport in the world. From its humble beginning in the 1971 Women’s World Cup, to a major spectacle today in both league and international tournaments, the sport is further diversified.^21^ The diversity of talented players, regardless of nationality, ethnicity or gender, enrichens the sport, improves the game, and heightens the spectacle of competition.

Medical teams function best when talented individuals with a diversity of backgrounds, ethnicities, and ideas work collectively toward shared goals. A diverse ID workforce enhances the triple aim of clinical excellence, workforce excellence, and health equity.^22^ In ID, a diverse and inclusive workforce Improves patient trust, communication, and engagement.^22^ Further, diversity enhances care for populations disproportionately affected by IDs.^23^ An inclusive ID workforce also strengthens team performance by fostering broader perspectives and reducing blind spots.^24^ As ID faces a shrinking workforce, increased diversity Improves recruitment, retention, and engagement of healthcare professionals.^24^ Last, promoting diversity increases representativeness and participation in IDs research and clinical trials, something that will benefit research missions and a greater patient population.^25^

## Lessons from defeat: do you play to win, or not to lose?

High level competitions are meant to be won, or so it seems. In the book *Thinking, Fast and Slow*, Kahneman and Tversky introduce the concept of what can occur to groups or individuals under pressure: loss aversion.^26^ In soccer terms, teams can play not to lose, instead of playing to win. Due to loss aversion, the threat of a possible loss feels bigger than the value of a possible win. In the 2026 World Cup semi-final match, England went up 1–0 over rival Argentina. With nearly 30 minutes left to play, England manager made some baffling decisions, substituting midfield and attacking players, to preserve a one goal lead against a storied Argentine team.^27^ These premature defensive changes shifted the momentum entirely to Argentina, ceded control of the ball to the opponent, and unlocked Lionel Messi, giving him the space and time to control the attack. The result was a 2–1 loss with the England proving incapable of stopping the two late game goals by Argentina. Instead of chasing the win with a second goal, Tuchel played not to lose, with disastrous consequences.

At the individual level, playing to win in clinical ID is consistent with advancing as a team, particularly in group-based compensation plans. In this scenario maximizing billing and coding opportunities in clinical care justifies new positions and financial growth for the division. Although at times it may be more convenient for individuals to see fewer patients and write less notes when fatigued, collectively, these measures go a long way. Conversely, positions can get cut or program expansion denied if we don’t demonstrate their financial viability within reasonable work standards.^5^

In leadership, playing not to lose produces decisions marked by risk aversion and the lack of a growth agenda. Conversely, when playing to win, IDs leaders set the agenda, particularly when facing a receding ID workforce and increased demand for our services. This requires leadership and advocacy at both institutional, regional and national levels to reinvest and reinforce the ID workforce. This necessitates a strategic framework to bolster the ID workforce by addressing low compensation through value-based pay models and loan repayment, while mitigating immigration barriers and political hostility. Along with my colleagues, we play to win and successfully restructured our compensation, advocated for protective legislative precedents for foreign-born physicians, and improving the public perception of the specialty through speaking out, publishing editorials, voting, and engaging peacefully.^28,29^

## Dutch total football is total teamwork—toward total ID consultation

The Netherlands is the greatest soccer nation to never win the World Cup, despite three appearances in the finals. Perhaps Dutch soccer is best known for the concept of Total Football, pioneered by legendary players Johan Cruyfff and Johan Neeskens along with manager Rinus Michels.^30^ Total Football is based on principle that no player, except the goalkeeper, should have a fixed position. Their roles are fluid, with individuals encouraged to frequently interchange to create devastating attacking patterns of play. The method requires high levels of football intelligence, technical ability, and communication, with players constantly needing to adopt new positions depending on their teammates’ movements. When done correctly, Total Football blends attacking fluency with intense, proactive defending. This philosophy and style of play led The Netherlands to multiple historic World Cup runs, and when exported by Johan Cruyff, later in the role of manager to FC Barcelona, resulted in successive Spanish League titles and the 1992 European Cup. Total football is total teamwork.

The busy, inpatient IDs consult service requires a collective mind set shift. In institutions with multiple parallel services such as general ID teaching services, general ID faculty services, transplant ID, musculoskeletal infections and telehealth consults, providers commonly function as siloed, individual consultants. This model requires an explicit philosophical and practical shift in both form and function. Rather than individual consultants functioning independently in their designated areas of interest, the goal is to flow like a *single team* with common goal of inpatient patient care. Like a sports team with individual positions, we have our individual clinical focus, but also like a sports team, we synergize and cover each other on the field of play (hospital floors). This is applicable if consult service volumes are inordinately high and unevenly distributed across services or if a team member is unexpectedly out owing to illness or family issues.

If any inpatient consult service requires help from another, regardless of the primary focus, the others are *obligated and expected* to assist. Recognizing that our skills are not fully transferable, and that within ID specialized knowledge exists, there is generally sufficient overlap to provide cross-coverage in the face of urgent or last-minute consult schedule disruptions. This requires communication and trust between all team members and a willingness to minimize martyrdom and ask for assistance.^5^ In the end, the result is the “Total ID Consult” team, one that maximizes flow, efficiency, and collaboration so that work hours are reasonable, individuals are engaged and feel collectively supported with a group output that is greater than the sum of the parts.

## Stoppage time

“I’m a rock star because I couldn’t be a footballer.”—Bono

**Figure 1.**
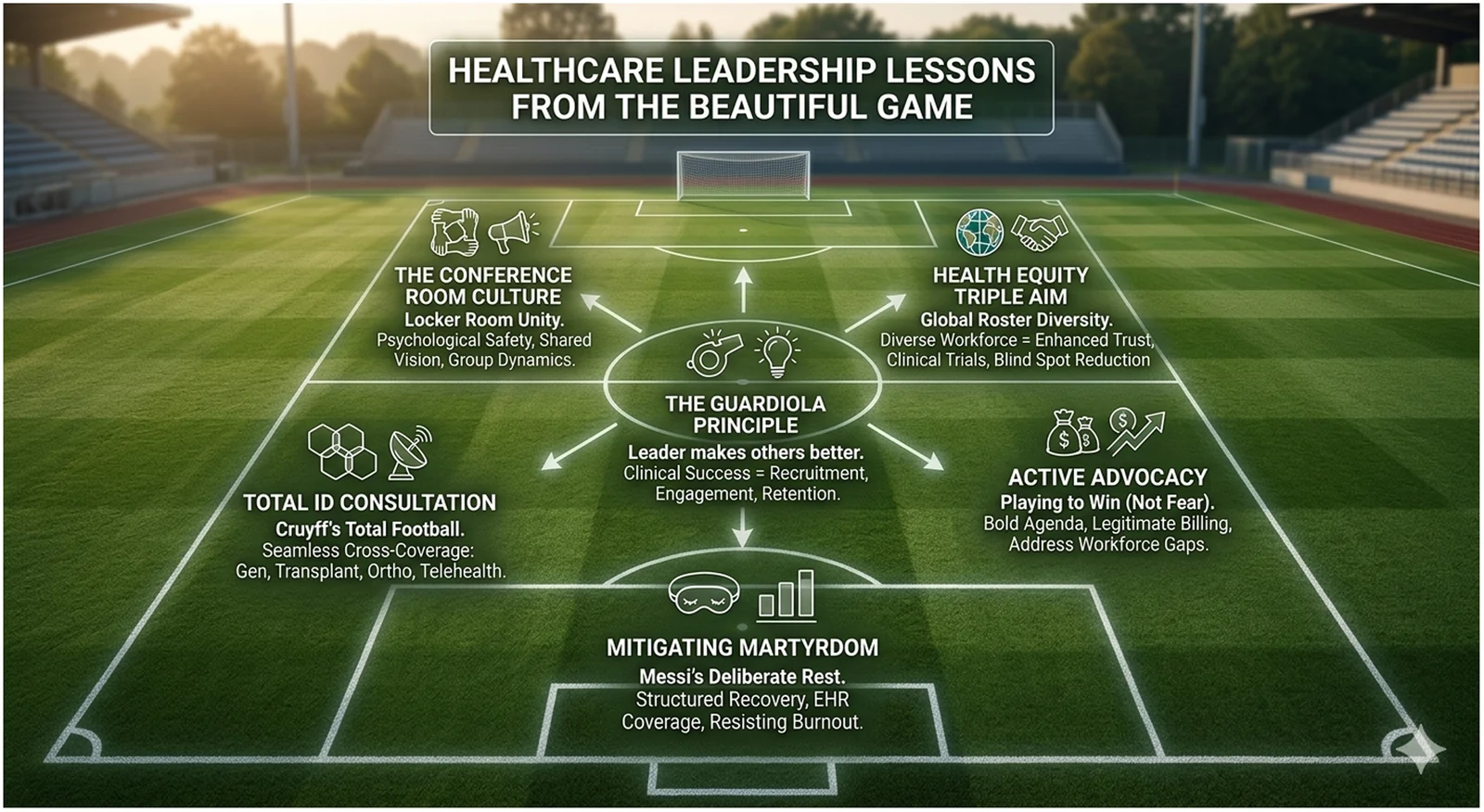
Figure 1 long description. Healthcare ledership lessons from the beautiful game. Disclaimer: Generative AI was utilized to create this figure from the contents of this manuscript. The author takes full responsibility for the content.

I am a doctor probably because I could not be a professional footballer. However, I continue to learn and find leadership inspiration from my favorite sport (Figure 1). The parallels between *O Jogo Bonito* and academic medicine offer a powerful metaphor for practice and leadership. Just as elite soccer requires a deliberate blend of passion, rigorous mentorship, and strategic energy conservation, effective clinical leadership depends on fostering the growth of others while actively protecting their well-being. The genius of Lionel Messi reminds us that peak performance is not sustained by continuous, relentless effort, but by mastering the art of deliberate rest and sharp situational awareness. By moving away from a culture of medical martyrdom and instead modeling structured recovery, clinical leaders can transform their teams from overextended individuals into a resilient, diverse, cohesive unit. Ultimately, whether on the pitch or at the bedside, the true measure of success lies not just in winning the immediate match or conquering the workday, but in taking calculated, growth-oriented risks and cultivating an environment where talent can endure, collaborate, and thrive for the long term.

## Figure 1. Long description

The diagram features a soccer field layout with various labeled sections representing different leadership principles. At the center, ‘The Guardiola Principle’ is highlighted, emphasizing leaders making others better. Surrounding this central concept are five key areas: ‘The Conference Room Culture’ focusing on unity and shared vision, ‘Total ID Consultation’ advocating for seamless cross-coverage in healthcare, ‘Health Equity Triple Aim’ promoting workforce diversity and trust, ‘Active Advocacy’ encouraging bold agendas and addressing workforce gaps, and ‘Mitigating Martyrdom’ stressing structured recovery and resisting burnout. Each section is depicted with relevant icons and brief descriptions, illustrating the parallels between soccer strategies and healthcare leadership.

Navigate back to Figure 1..
